# A sexual health quality improvement program (SHIMMER) triples chlamydia and gonorrhoea testing rates among young people attending Aboriginal primary health care services in Australia

**DOI:** 10.1186/s12879-015-1107-5

**Published:** 2015-09-02

**Authors:** Simon Graham, Rebecca J. Guy, Handan C. Wand, John M. Kaldor, Basil Donovan, Janet Knox, Debbie McCowen, Patricia Bullen, Julie Booker, Chris O’Brien, Kristine Garrett, James S. Ward

**Affiliations:** Kirby Institute, UNSW Australia, Sydney, Australia; Centre for Epidemiology and Biostatistics, School of Population and Global Health, University of Melbourne, Melbourne, Australia; Sydney Sexual Health Centre, Sydney Hospital, Sydney, Australia; Aboriginal Community Controlled Health Service, Sydney, New South Wales Australia; South Australian Health and Medical Research Institute, Adelaide, Australia

## Abstract

**Background:**

In Australia, chlamydia is the most commonly notifiable infection and over the past ten years chlamydia and gonorrhoea notification rates have increased. Aboriginal compared with non-Aboriginal Australians have the highest notifications rates of chlamydia and gonorrhoea. Regular testing of young people for chlamydia and gonorrhoea is a key prevention strategy to identify asymptomatic infections early, provide treatment and safe sex education. This study evaluated if a sexual health quality improvement program (QIP) known as SHIMMER could increase chlamydia and gonorrhoea testing among young people attending four Aboriginal primary health care services in regional areas of New South Wales, Australia.

**Methods:**

We calculated the proportion of 15–29 year olds tested and tested positivity for chlamydia and gonorrhoea in a 12-month before period (March 2010-February 2011) compared with a 12-month QIP period (March 2012-February 2013). Logistic regression was used to assess the difference in the proportion tested for chlamydia and gonorrhoea between study periods by gender, age group, Aboriginal status and Aboriginal primary health service. Odds ratios (OR) and their 95 % confidence intervals (CIs) were calculated with significance at *p* < 0.05.

**Results:**

In the before period, 9 % of the 1881 individuals were tested for chlamydia, compared to 22 % of the 2259 individuals in the QIP period (OR): 1.43, 95 % CI: 1.22-1.67). From the before period to the QIP period, increases were observed in females (13 % to 25 %, OR: 1.32, 95 % CI: 1.10-1.59) and males (3 % to 17 %, OR: 1.85, 95 % CI: 1.36-2.52). The highest testing rate in the QIP period was in 15–19 year old females (16 % to 29 %, OR: 1.02, 95 % CI: 0.75-1.37), yet the greatest increase was in 20–24 year olds males (3 % to 19 %, OR: 1.65, 95 % CI: 1.01-2.69). Similar increases were seen in gonorrhoea testing. Overall, there were 70 (11 %) chlamydia diagnoses, increasing from 24 in the before to 46 in the QIP period. Overall, 4 (0.7 %) gonorrhoea tests were positive.

**Conclusions:**

The QIP used in SHIMMER almost tripled chlamydia and gonorrhoea testing in young people and found more than twice as many chlamydia infections. The QIP could be used by other primary health care centres to increase testing among young people.

## Background

Chlamydia is the most commonly notified infectious disease in Australia, the United States of America (US) and the most commonly reported sexually transmissible infection (STI) among 16–24 year olds in the United Kingdom (UK) [1–3]. In Australia, the highest chlamydia notification rates are in females, 15–29 year olds and young Aboriginal and Torres Strait Islander (hereafter referred to as ‘Aboriginal’) people living in non-urban areas [1, 4]. In 2013, notification rates among Aboriginal compared with non-Aboriginal people in Australia were three times greater for chlamydia and 14 times greater for gonorrhoea [1].

In 2011, the Australian Bureau of Statistics (ABS) estimated that there were 669881 Aboriginal people, which accounts for approximately 3 % of the Australian population [5]. The number of Aboriginal people varied across the Australian jurisdictions with the largest residing in New South Wales (208476) followed by Queensland (188954) [5]. The median age of Aboriginal people was 21 years compared with 37 years in non-Aboriginal people [6], with a higher proportion of Aboriginal compared with non-Aboriginal people living in remote areas [7]. Aboriginal compared with non-Aboriginal people have higher rates of chronic and communicable diseases and unemployment; and lower levels of home ownership, school completion and life expectancy [7]. As a result, the Australian government has released a number of national strategies to reduce the disparity between Aboriginal and non-Aboriginal people, including the *Fourth National Aboriginal and Torres Strait Islander Blood Borne Viruses and Sexually Transmissible Infection Strategy, 2014–2017* [8].

Chlamydia is predominantly asymptomatic [9], and gonorrhoea is mostly asymptomatic in females and in about half of males [9]. This highlights that testing is an important prevention strategy to identify infections early, provide treatment, prevent poor reproductive health outcomes from developing and prevent transmitting the infections to others. If left untreated, chlamydia and gonorrhoea infection can lead to pelvic inflammatory disease (PID) [10], ectopic pregnancy [11] and infertility [12].

STI guidelines in the US and UK recommend annual chlamydia testing in 16–24 year old females [2, 3], and Australian STI guidelines recommend annual chlamydia testing among sexually active 15–29 year old females and males [13]. Australian STI guidelines for Aboriginal people recommend gonorrhoea testing in 15–39 year olds where the prevalence is high [14], for example in remote areas of Australia [1]. Also improvements in laboratory testing has influenced gonorrhoea testing in Australia with a recent study finding that between 2007 to 2012 there has been a 32 % increase in laboratories using duplex nucleic acid amplification (NAAT) to test for chlamydia and gonorrhoea [15]. NAAT can test urine or swab specimens for chlamydia and gonorrhoea at the same time, even if a test for only one organism was requested.

In Australia, it is estimated that 85 % of females and 64 % of males aged 16–29 years attend a primary health care service annually for their health care needs [16]. Aboriginal Community Controlled Health Services (ACCHS) are primary health care centres which provide culturally appropriate medical, allied health and health education to Aboriginal people [17]. There are an estimated 142 ACCHS in Australia and 42 in New South Wales (NSW) [17].

Over the past decade, a number of interventions have been trialed in primary health care centres that have aimed to increase STI testing, including quality improvement programs (QIP) [18, 19]. QIP involves collecting data, visiting the health centre regularly, presenting the results to clinic staff and facilitating a discussion with staff to develop strategies which could improve clinical management of an infection and monitor the clinics performance over time. A study conducted in paediatric clinics in the US used a QIP process to increase the proportion of 14–18 year olds tested for chlamydia [20]. The study increased the proportion of males tested for chlamydia from 0 to 60 % in QIP sites compared with 0 to 5 % in control sites. This was achieved through providing training, developing testing protocols and reviewing these protocols every month with the clinic team and discussing ways to reduce barriers to testing and ways to actively increase testing. In Australia, a sexual health intervention was introduced in primary health care centres which used a similar QIP process described in the US study [21]. The intervention was able to increase chlamydia testing rates from 6 to 10 % in females and from 4 to 6 % in males. The project in Australia also conducted interviews with clinic staff to examine what strategies could further increase testing rates. Responses included, financial incentives for physicians, health promotion targeting young people to increase patient demand for testing and ensuring quality data is provided to monitor the clinic’s performance and provide regular feedback to clinic staff [21].

This study evaluates the impact of a sexual health QIP on chlamydia and gonorrhoea testing rates and positivity among 15–29 year olds attending four ACCHS in regional areas of NSW.

## Methods

### Setting

This study was conducted in the context of a sexual health QIP called SHIMMER. SHIMMER was conducted in collaboration with four ACCHS located in regional areas of NSW, Australia [22–24]. The ACCHS varied in size and scope of medical, allied health and health education services to local Aboriginal communities.

### Quality improvement program

The QIP used in SHIMMER was adapted from another QIP project known as STRIVE (*STIs in Remote communities: ImproVed & Enhanced primary health care trial)* [25]. STRIVE is a cluster randomised control trial in 65 remote Aboriginal primary health care centres in Northern Australia. The aim of STRIVE was to decrease the prevalence of chlamydia, gonorrhoea and trichomonas among 16–34 year olds through a QIP process that aims to increase testing and improve the management of these infections. The QIP process in STRIVE involved;

1. The extraction of attendance and STI testing data from the electronic patient system;
2. Developing indicators based on local STI guidelines and producing reports based on these indicators;
3. Providing monetary incentives if an increase in testing rates were achieved;
4. Using a systems assessment tool to review their STI program more broadly;
5. A coordinator (nurse or sexual health physician) visiting the Aboriginal primary health care centres every 6-months to present the STI reports and facilitating a conversation with clinic staff to develop STI testing and management strategies and document these into an action plan.

SHIMMER involved a similar QIP process to STRIVE, except;

1. A sexual health physician and project manager visited the ACCHS more frequently (every four to six months (a total of five visits per ACCHS over a two year period);
2. The indicators were based on national STI guidelines;
3. SHIMMER provided an annual payment to each ACCHS that was not performance based; and
4. A clinical audit was conducted to examine the reasons why 15–24 year olds attended the ACCHS and what types of consultations (described in the statistical analysis section) included chlamydia and gonorrhoea testing.

The STI testing and management strategies developed by the ACCHS staff during the visits were specific to each ACCHS to maximise the success of the strategies to increase STI testing rates (Table 1). The project manager was an Aboriginal man who co-delivered the intervention with a sexual health physician. The ethical conduct of SHIMMER was guided by the Australian National Health and Medical Research Council *Guidelines for Ethical Conduct in Aboriginal and Torres Strait Islander Health Research* [26]. This document strongly encourages research projects to have a project reference group where Aboriginal people are active members in all aspects of the research including the design, implementation, and interpretation of the results [26]. Peer-based interventions and health promotion is a well-accepted means of reaching marginalised communities. In the US, breast cancer programs delivered by Native American health workers to Native American communities increased the recruitment of Native Americans in the program, increased breast cancer screening, re-screening, and decreased perceived barriers to screening. This study also observed increases in knowledge regarding Pap tests, cervical cancer and awareness of cervical cancer issues [27, 28].

**Table 1:** Examples of STI testing and management strategies used by the ACCHS

Goal	Strategy	Who
To increase chlamydia & gonorrhoea testing	Add chlamydia & gonorrhoea testing to the adult health check^a^ template	Practice manager
	Offer more Adult Health Checks to 15–29 year olds	All clinic staff
	Offer testing to any females or male aged 15–29 years	All clinic staff
	Offer testing to any female aged 15–29 years who attends for a Pap smear, a pregnancy test, contraception or antenatal care	All clinic staff
	Place laminated reminders to test 15–29 year olds near urine pots kept in consult rooms and stock room. Attached laminated reminder to test where pregnancy kits are kept	Practice manager
	Place laminated reminder to test next to contraceptive devices (e.g. Implanon, contraceptive pills)	
Improve STI re-testing	Anyone who tests positive for chlamydia and/or gonorrhoea - enter recall into patient system for a re-test 3-months after treatment	All clinic staff
	Anyone aged 15–29 years who tests negative - Enter recall into patient system for re-test in 12 months.	
	Place laminated signs in treatment cupboard to enter recall into patient system for a re-test 3 months after treatment of chlamydia or gonorrhoea	

^a^Adult health check is a general health assessment used by ACCHS

### Evaluation design

A before and after study design was used. Attendance and STI testing data from the ACCHS was used to calculate the proportion of 15–29 year olds tested and tested positive for chlamydia and gonorrhoea, during a 12-month before period (March 2010 to February 2011) compared with a 12-month QIP period (March 2012 to February 2013). We chose the 12-month before period because during the 12-months prior to the QIP period (March 2011 to February 2012) the SHIMMER researchers were in discussions with each ACCHS to establish the project and clinic staff knew that a sexual health QIP was going to occur, which potentially could have influenced staff offering more STI testing as result of these initial project discussions. As a result the chosen 12-month before period provides a more accurate baseline period that is not influenced by the establishment of the SHIMMER project. We also conducted a clinical audit to determine why 15–24 year olds were attending the ACCHS and the type of consultations where chlamydia and gonorrhoea testing occurred during a six-month before period (1^st^ July to 31^st^ December 2011) compared with a six-month QIP period (1^st^ July to 30^th^ November 2012).

### Data sources

1. Attendance, testing and positivity data
  We used a data extraction tool called ‘GeneRic Health Network Information Technology for the Enterprise’ (GRHANITE^TM^) [29] to extract attendance and STI testing data on a weekly basis through the internet from the electronic patient system of each ACCHS. The software was installed just prior to the QIP commencing and the first extraction included two years of retrospective data. Data included a unique de-identified patient number, gender, age, Aboriginal status, practitioner type (physician, nurse or dentist), consultation date, chlamydia and gonorrhoea testing and result.
2. Clinical audit
  A sexual health physician conducted a clinical audit of 100 individuals aged 15–24 years who attended in a six-month before period and a six-month QIP period. Attendees were sorted by name, and all consultations of the first 50 females and 50 males in the two time periods were reviewed. The audit focused on a younger age group compared with GRHANITE^TM^ for two reasons. Firstly, the audit was resource intensive (a week at each ACCHS) and secondly, over the past decade chlamydia positivity has been highest among 15–24 year olds. Data extracted included a de-identified patient number, gender, age, consultation date, consultation type (the reason they attended the ACCHS), if a chlamydia and gonorrhoea test occurred, test results and potential STI symptoms. Two of the ACCHS did not have a total of 50 males who attended in both the six-month before period or the six-month QIP period and as a result the total number of individuals in the before and QIP periods are not the same.

### Statistical analysis

Using the GRHANITE™ data, the proportion of 15–29 year olds tested for chlamydia and gonorrhoea was calculated as follows; in each 12-month period, the number of individuals tested divided by the number of individuals who attended (repeat tests and visits of the same person were removed). The proportion of 15–29 year olds testing positive for chlamydia and gonorrhoea was calculated as follows; in each 12-month period, the number of individuals who tested positive divided by the number of individuals who were tested (repeat positives and tests of the same person were removed).

Using the clinical audit data, the proportion of 15–24 year olds tested for chlamydia and gonorrhoea by consultation type was calculated as follows; in each six-month period, the number of individuals tested by the number of individuals who attended (repeat tests and visits of the same person were removed). The consultation types were divided into six categories; *STI symptom/check* (STI check, discharge, dysuria), *reproductive health* (contraception, pap smear, pregnancy test); *adult health check (*a general health assessment which includes around ten clinical assessments/tests and is recommended annually to 15–54 year old Aboriginal people, each ACCHS decides which tests are included in their template*)*; *general medical* (ear, nose and throat, skin, respiratory tract infection, urinary tract infection, back pain, migraines, thyroid problems, referrals, vaccinations, smoking cessation); *mental health* (depression, anxiety, suicidal thoughts); and *injury* (broken bones, bruises). As people can attend for multiple reasons, we created a hierarchy in the order above. For example, if a consultation included a discussion about STI symptoms/check and mental health, it was identified as a STI symptom consultation. In both data sources, allied health consultations such as dental and physiotherapy were excluded as these practitioners cannot offer STI testing, treatment and education.

A chi-squared test was used to assess if patient demographics (gender, age group, Aboriginal status and ACCHS) and the proportion of 15–29 year olds ever tested for chlamydia and gonorrhoea were significantly different in the 12-month before period compared with the 12-month QIP period. Logistic regression was used to assess if the proportion tested for chlamydia and gonorrhoea was significantly different in the before period compared with the QIP period by gender, age group and Aboriginal status. Odds ratios (OR) and their 95 % confidence intervals (95 % CI) were calculated with statistical significance at *p* < 0.05. Data were analysed using STATA 12 statistical software (STATA Corporation, College Station TX) and Microsoft Excel 2007.

Ethical approval was received from the Aboriginal Health & Medical Research Council of NSW Ethics Committee, the UNSW Australia Human Research Ethics Committee and the Boards of each participating ACCHS. The SHIMMER project was funded by the NSW Ministry of Health.

## Results

### Attendance

Using the GRHANITE™ data, there were 1881 individuals aged 15–29 years who attended in the before period and 2259 in the QIP period. In the audit, there were 408 individuals aged 15–24 years in the before period and 437 in the QIP period.

### Testing rates

Using the GRHANITE™ data, the proportion tested for chlamydia in the before period was 9 %, increasing to 22 % (Table 2) in the QIP period (OR: 1.43, 95 % CI: 1.22-1.67). In the QIP period, a higher testing rate was achieved in females; increasing from 13 % to 25 % (OR: 1.32, 95 % CI: 1.10-1.59). However the increase was greater in 20–24 year old males due to the lower testing rates in the before period; increasing from 3 % to 17 % (OR: 1.65, 95 % CI: 1.01-2.69, Table 2). Among females, the highest testing rate achieved in the QIP period was in 15–19 year olds, increasing from 16 % to 29 % (OR: 1.02, 95 % CI: 0.75-1.37). Testing rates varied in the four ACCHS sites, with three showing significant increases between the before and QIP periods and one having moderate increases. ACCHS site 1 achieved the highest testing rates in 15–29 year olds, increasing from 10 % to 40 % in females and from 2 % to 27 % in males (Table 2). Similar increases were observed for gonorrhoea testing (Table 2).

**Table 2:** Proportion tested for chlamydia and gonorrhoea in the baseline compared with the quality improvement program (QIP) period by demographic factors and ACCHS site

	Chlamydia testing			Gonorrhoea testing		
	Period			Period		
Demographic factors & ACCHS site	Baseline	QIP^a^	*P*-value^b^	Baseline	QIP^a^	*P*-value^b^
	n (%)	n (%)		n (%)	n (%)	
Overall	175 (9)	492 (22)	<0.001	116 (6)	461 (20)	<0.001
Females						
Overall	150 (13)	335 (25)	<0.001	94 (7)	306 (23)	<0.001
Age group (years)						
15-19	61 (16)	132 (29)	<0.001	34 (9)	121 (27)	<0.001
20-24	59 (14)	119 (24)		43 (10)	110 (22)	
25-29	30 (9)	84 (21)		17 (5)	75 (19)	
Aboriginal status						
Aboriginal	133 (14)	291 (27)	<0.001	83 (9)	266 (25)	<0.001
Non-Aboriginal	17 (8)	43 (15)		11 (5)	39 (14)	
ACCHS site^c^						
1	13 (10)	74 (40)	<0.001	0 (0)	69 (37)	<0.001
2	104 (22)	149 (32)		73 (15)	137 (29)	
3	9 (6)	28 (7)		7 (3)	22 (5)	
4	24 (8)	84 (29)		14 (5)	78 (27)	
Males						
Overall	25 (3)	157 (17)	<0.001	22 (3)	155 (17)	<0.001
Age group (years)						
15-19	11 (4)	58 (17)	<0.001	10 (3)	58 (17)	<0.001
20-24	7 (3)	56 (19)		5 (2)	55 (19)	
25-29	7 (3)	43 (15)		7 (2)	42 (14)	
Aboriginal status						
Aboriginal	24 (4)	140 (18)	<0.001	21 (3)	138 (18)	<0.001
Non-Aboriginal	1 (1)	17 (11)		1 (1)	17 (11)	
ACCHS site^c^						
1	3 (2)	42 (27)	<0.001	0 (0)	42 (27)	<0.001
2	20 (7)	68 (17)		20 (7)	65 (17)	
3	1 (1)	15 (7)		1 (1)	16 (7)	
4	1 (1)	32 (19)		1 (1)	32 (19)	

^a^QIP (quality improvement program)

^b^Pearson χ2 test (significance *p* < 0.05)

^c^Aboriginal Community Controlled Health Service

### Consultation types

In the clinical audit, chlamydia testing in the before period was most commonly conducted when patients presented with STI symptoms/check, reproductive health, or adults health checks, but these types of consultations only accounted for a combined total of 31 % of total consultations; with general medical consultations accounting for the largest proportion of consultations (56 %). Among females, the proportion tested for chlamydia increased during the following consultation types; STI symptom/checks (from 65 % in the before to 80 % in the QIP period, adult health check (50 % to 62 %) and reproductive health (29 % to 39 %) consultations (Table 3). Among males, the proportion tested for chlamydia increased during STI symptom/checks (67 % to 70 %), adult health check (50 % to 75 %) and mental health (14s % to 37 %) consultations (Table 3). Similar increases were observed for gonorrhoea testing however the relative increase was greater as fewer gonorrhoea tests were conducted during the before period compared with chlamydia (Table 3).

**Table 3:** Proportion tested for chlamydia and gonorrhoea in the baseline compared with the quality improvement program (QIP) audit by consultation type

Patient gender & consultation types	Consultations		Chlamydia testing			Gonorrhoea testing		
	Period		Period			Period		
	Baseline	QIP^a^	Baseline	QIP^a^	*p*-value^b^	Baseline	QIP^a^	*p*-value^b^
	n (%)	n (%)	n (%)	n (%)		n (%)	n (%)	
Overall	408 (48)	437 (52)	95 (23)	109 (25)	0.573	49 (12)	107 (24)	<0.001
Females								
Overall	248 (61)	226 (52)	58 (23)	68 (30)	0.099	34 (14)	64 (28)	<0.001
Consultations types								
General medical^c^	140 (56)	104 (46)	19 (14)	12 (12)	0.637	11 (8)	11 (11)	0.463
STI symptom/ check^d^	20 (8)	25 (11)	13 (65)	20 (80)	0.258	8 (40)	21 (84)	0.002
Reproductive Health^e^	55 (22)	61 (27)	16 (29)	24 (39)	0.246	9 (16)	21 (34)	0.027
Adult Health Check^f^	12 (5)	16 (7)	6 (50)	10 (62)	0.508	3 (25)	10 (62)	0.049
Mental health^g^	17 (7)	13 (6)	3 (18)	2 (15)	0.869	3 (18)	1 (8)	0.238
Injury^h^	4 (2)	7 (3)	1 (25)	0 (0)	0.165	0 (0)	0 (0)	N/A
Males								
Overall	159 (39)	210 (48)	37 (23)	41 (19)	0.387	15 (9)	43 (20)	0.004
Consultations types								
General medical^c^	95 (59)	132 (62)	14 (15)	8 (6)	0.029	5 (5)	8 (6)	0.788
STI symptom/ check^d^	9 (5)	10 (5)	6 (67)	7 (70)	0.876	4 (44)	7 (70)	0.260
Adult Health Check^f^	26 (16)	24 (11)	13 (50)	18 (75)	0.069	4 (15)	19 (79)	0.053
Mental health^g^	14 (8)	19 (9)	2 (14)	7 (37)	0.150	2 (14)	7 (37)	0.150
Injury^h^	15 (9)	25 (12)	2 (13)	1 (4)	0.278	0 (0)	2 (8)	0.261

^a^QIP (quality improvement program)

^b^Pearson χ2 test (significance p < 0.05)

^c^General medical (ear, nose and throat, skin, respiratory tract infection, urinary tract infection, back pain, migraines, thyroid problems, referrals, vaccinations, smoking cessation)

^d^STI symptom/check (STI check, discharge, dysuria)

^e^Reproductive health (Pap smear, contraception, antenatal)

^f^Adult Health Check (a general health assessment which is recommended annually to 15-54 year old Aboriginal people)

^g^Mental Health (depression, anxiety, suicidal thoughts) h Injury (broken bones, bruises)

### Positivity

Using the GRHANITE™ data, there were 70 (11 %) chlamydia diagnoses, increasing from 24 in the before period to 46 in the QIP period. In females, the number of chlamydia diagnoses increased from 18 to 33 and in males from 6 to 13. The highest number of chlamydia diagnoses for both females and males occurred in 15–19 year olds (71 %), 35 for females and 15 for males. There were 577 gonorrhoea tests conducted over both periods with 4 (0.7 %) testing positive.

## Discussion

This study highlights that SHIMMER was able to significantly increase the proportion of 15–29 year olds tested for chlamydia and gonorrhoea. The increase in testing predominantly occurred during STI symptoms/check, reproductive health and adult health check consultations. However an increase in testing did not occur during general medical consultations, which provided the most opportunities to increase testing. Double the number of chlamydia diagnoses were detected during the QIP period compared with the before period and over the whole study period there were only four gonorrhoea diagnoses.

The main strength of SHIMMER was that we were able to collect a large sample size of 15–29 year olds and collect data from two sources. The clinical audit provided contextual information on why 15–24 year olds attended the ACCHS and the consultation types where testing had occurred. There are a few limitations to consider when interpreting our findings. Firstly, only four ACCHS participated in SHIMMER, as a result our results may not be generalisable to all ACCHS in NSW or Australia. Secondly, our study was based on attendance and testing data that was entered into the electronic patient records system, therefore if consultations or testing were not entered into the system it may have resulted in our study under-estimating the testing rates and positivity in the ACCHS. Thirdly, it is possible that young people could have also been tested for STIs at other health care centres in these regional towns, which would result in our study under-estimating the testing rates. However we do not think this would have been common as other studies have demonstrated that young Aboriginal people prefer to attend ACCHSs for STI testing [30]. A recent study included data from six ACCHS and 25 general practice centres in Australia and highlighted that only 1 % of 16–29 year olds who attended general practice centres were Aboriginal compared with 85 % who attended the ACCHS [31]. The above mentioned study also highlighted that the chlamydia testing rates among the young Aboriginal attendees were 20 % at ACCHS and 4 % at general practice centres [31]. Fourthly, we could not determine if the 15–29 year olds were local residents or visitors from other Aboriginal communities. If we were able to focus on just local residents the proportion of 15–29 year olds tested in this study may have been higher. It is acknowledged that the repeat visits by the sexual health physician and the project manager could have impacted upon the testing rates as the ACCHS clinic staff knew they would return on a regular basis to monitor their progress. Finally, our evaluation focused on the impact of the QIP on STI testing rates and did not involve a process or economic evaluation.

Overall the proportion of 15–29 year olds tested for chlamydia during the QIP period was 22 %, which is almost three-times greater than the 9 % in the before period. However one ACCHS in this study was able to increase their testing rate to 40 % among 15–29 year old females. The varying impact seen among ACCHS highlights two factors, that various factors can influence the success of the QIP process on testing rates in different ACCHS and the need to tailor QIP to each ACCHS as they have different staffing levels, resources, outreach and training levels. Although the 22 % in our study was lower than the 45 % achieved in US paediatric clinics [20], the magnitude of change in our program is similar to the US program due to the lower levels of testing among ACCHS in the 12-month before period. Also SHIMMER included a larger age group, and as a result demonstrated that the QIP was beneficial in the age group recommended by Australian STI testing guidelines for Aboriginal people [32]. During the QIP period in our study, 25 % of females and 17 % of males were tested for chlamydia, which was greater than the other QIP program in Australia implemented in regional primary health care centres in NSW, which resulted in 10 % of females being tested, with the increase predominantly occurring during consultations that included a Pap smear [21].

In SHIMMER, higher chlamydia testing rates were found among young people attending for STI symptoms/checks, reproductive health consultations and adult health checks (AHC). For every completed AHC the ACCHS can receive $212 from the Australian government’s Medicare Benefits Scheme. A completed AHC can be conducted and charged every nine-months [33]. If STI testing is added to the AHC template and more AHC are offered to young people, this may increase testing rates. This study also highlighted that not all STI symptoms/check consultations included a chlamydia and /or gonorrhoea test. This highlights the asymptomatic nature of chlamydia and gonorrhoea and if mild symptoms are present, these can be misinterpreted as other conditions. This also highlights the need for clinic staff to receive STI training so they are more aware of the mild symptoms of chlamydia and gonorrhoea. However the most effect strategy would be to base testing on the age of attendees to account for those with asymptomatic infection.

Based on the findings from this evaluation, the following strategies could be considered to increase testing further; initiatives to integrate testing into general medical consultations; offering more AHC to young people; and health promotion programs that encourage young people to present more frequently to the ACCHS and request STI testing or a combination of both. This is supported by an analysis of baseline data from SHIMMER which highlighted that chlamydia testing was strongly associated with those 15–29 year olds who attended the ACCHS more frequently compared with those who attended once to twice over a three-year period (OR:16.97) [22].

This study showed that as testing increased so did the number of chlamydia diagnoses. The highest chlamydia positivity was among 15–24 year olds which is consistent with national surveillance in Australia [1] and an analysis of chlamydia infections conducted in Aboriginal primary health care centres in remote areas of Northern Australia [34]. We found gonorrhoea positivity was very low over both periods. As gonorrhoea prevalence can fluctuate quickly [35], it is important to achieve high levels of gonorrhoea and chlamydia testing so that any infection is detected early and treatment provided. The increasing use of NAAT equipment by laboratories in Australia to test for chlamydia and gonorrhoea at the same time will assist in achieving higher testing rates of gonorrhoea [15].

Some aspects of the quality improvement process used in SHIMMER could be used in other primary health care settings (visits, reports, feedback), as currently being utilised in the Australian Chlamydia Control Effectiveness Pilot (ACCEPt) project – a trial of chlamydia screening in general practice clinics [36]. However some of the STI testing strategies developed by the ACCHS in SHIMMER are specific to Aboriginal people, such as adult health checks, which saw an increase in the testing rates however these are not offered in non-Aboriginal general practice clinics. The other testing and management strategies listed in Table 1 could be used to increase STI testing and improve the management of STIs among young people attending general practice clinics.

## Conclusions

This evaluation of the SHIMMER project demonstrated that a sexual health QIP was able to significantly increase chlamydia and gonorrhoea testing in young Aboriginal people and detect more infections. The strategies developed and implemented by the ACCHSs staff aimed to become routine clinical practice that could result in sustainable long term changes. The QIP process used in SHIMMER could be introduced in other primary health care centres to improve the testing and management of other infection/ conditions.

## Abbreviations

Aboriginal: Aboriginal and Torres Strait Islander
ACCHS: Aboriginal Community Controlled Health Services
AH&MRC: Aboriginal Health & Medical Research Council
ABS: Australian Bureau of Statistics
BBV: Blood borne viruses
CIs: 95 % confidence intervals
GRHANITE^TM^: Generic Health Network Information Technology for the Enterprise
NSW: New South Wales
OR: Odds ratios
PID: Pelvic inflammatory disease
QIP: Quality improvement program
STI: Sexually transmissible infection
STRIVE: STIs in Remote communities: Improved & Enhanced primary health care trial
SHIMMER: Sexual health quality improvement program
UNSW: University of New South Wales
UK: United Kingdom
US: United States of America

## References

[1] Kirby Insitutue. Bloodborne viral and sexually transmitted infections in Aboriginal and Torres Strait Islander people: Annual Surveillance Report 2014. Kirby Institute, UNSW Australia. http://kirby.unsw.edu.au/sites/default/files/hiv/resources/2014ATSIP-ASR.pdf. Accessed on 30 April 2015.

[2] Centers for Disease Control and Prevention. 2013 Sexually Transmitted Diseases Surveillance. National Center for HIV/AIDS, Viral Hepatitis, STD and TB Prevention, Centers for Disease Control and Prevention; 2014. http://www.cdc.gov/std/stats13/. Accessed on 27 July 2015.

[3] Public Health England. Infection report: Sexually transmitted infections and chlamydia screening in England. Health Protection Report, Public Health England; 2014; vol 8. https://www.gov.uk/government/uploads/system/uploads/attachment_data/file/326935/hpr2414.pdf. Assessed on 27 July 2015.

[4] Graham S, Guy RJ, Donovan B, McManus H, Su JY, El-Hayek C (2012). Epidemiology of chlamydia and gonorrhoea among Indigenous and non-Indigenous Australians, 2000–2009. Med J Aust.

[5] Australian Bureau of Statistics. Estimates of Aboriginal and Torres Strait Islander Australian. June 2011. Australian Bureau of Statistics. http://www.abs.gov.au/ausstats/abs@.nsf/mf/3238.0.55.001. Assessed on 20 April 2015.

[6] Australian Bureau of Statistics. Aboriginal and Torres Strait Islander Population. 2012 year book Australia: a comprehensive source of information about Australia. Australian Bureau of Statistics; 2012. http://www.abs.gov.au/ausstats/abs@.nsf/Lookup/by%20Subject/1301.0~2012~Main%20Features~Aboriginal%20and%20Torres%20Strait%20Islander%20population~50. Accessed 20 April 2015.

[7] Australian Institute of Health and Welfare. Determinents of health and welfare. The Health and Welfare of Australia’s Aboriginal and Torres Strait Islander Peoples 2011. Australian Institute of Health and Welfare. http://aihw.gov.au/indigenous-observatory/health-and-welfare/. Accessed 20 April 2015.

[8] Commonwealth of Australia’s Department of Health and Ageing. BBV and STI in Aboriginal and Torres Strait Islander People. Fourth National Aboriginal and Torres Strait Islander Blood-borne Viruses and Sexually Transmissible Infections Strategy, 2014–2017. Department of Health and Ageing, Canberra; 2014. http://www.health.gov.au/internet/main/publishing.nsf/Content/ohp-bbvs-atsi. Accessed 20 April 2015.

[9] Korenromp EL, Sudaryo MK, de Vlas SJ, Gray RH, Sewankambo NK, Serwadda D (2002). What proportion of episodes of gonorrhoea and chlamydia becomes symptomatic?. Int J STD AIDS.

[10] Paavonen J (1980). Chlamydia-Trachomatis in Acute Salpingitis. Am J Obstet Gynecol.

[11] Westrom L, Bengtsson LP, Mardh PA (1981). Incidence, trends, and risks of ectopic pregnancy in a population of women. Br Med J (Clin Res Ed).

[12] Westrom L, Joesoef R, Reynolds G, Hagdu A, Thompson SE (1992). Pelvic inflammatory disease and fertility. A cohort study of 1844 women with laparoscopically verified disease and 657 control women with normal laparoscopic results. Sex Transm Dis.

[13] Australasian Sexual Health Alliance. Chlamydia and gonorrhoea management and treatment. Australian STI Management Guidelines for use in primary care. 2014. http://www.sti.guidelines.org.au/. Accessed 20 April 2015.

[14] National Aboriginal Community Controlled Health Organisation and the Royal Australian College of General Practitioners. Sexual health and bloodborne viruses. National guide to a preventative health assessment for Aboriginal and Torres Strait Islander people, 2nd edition. National Aboriginal Community Controlled Health Organisation and the Royal Australian College of General Practitioners. 2012. http://www.naccho.org.au/download/aboriginalhealth/1.National%20guide%20to%20a%20preventative%20health%20assessment%20for%20Aboriginal%20and%20Torress%20Strait%20Islander%20people%20(2).pdf. Accessed 20 April 2015.

[15] Donovan B, Dimech W, Ali H, Guy R, Hellard M (2015). Increased testing for Neisseria gonorrhoeae with duplex nucleic acid amplification tests in Australia: implications for surveillance. Sex Health.

[16] Kong FY, Guy RJ, Hocking JS, Merritt T, Pirotta M, Heal C (2011). Australian general practitioner chlamydia testing rates among young people. Med J Australia.

[17] Aboriginal Health & Medical Research Council of New South Wales. Who we are: Annual Report 2013–2014. Aboriginal Health & Medical Research Council of New South Wales. 2014. http://www.ahmrc.org.au/index.php?option=com_docman&task=cat_view&gid=29&Itemid=45. Accessed 20 April 2015.

[18] Guy RJ, Ali H, Liu B, Poznanski S, Ward J, Donovan B (2011). Efficacy of interventions to increase the uptake of chlamydia screening in primary care: a systematic review. BMC Infect Dis.

[19] Bailie RS, Si D, O’Donoghue L, Dowden M (2007). Indigenous health: effective and sustainable health services through continuous quality improvement. Med J Aust.

[20] Tebb KP, Pantell RH, Wibbelsman CJ, Neuhaus JM, Tipton AC, Pecson SC (2005). Screening sexually active adolescents for Chlamydia trachomatis: what about the boys?. Am J Public Health.

[21] Merritt TD, Durrheim DN, Hope K, Byron P (2007). General practice intervention to increase opportunistic screening for chlamydia. Sex Health.

[22] Graham S, Wand HC, Ward JS, Knox J, McCowen D, Bullen P, et al. Attendance patterns and Chlamydia and gonorrhoea testing among young people in Aboriginal primary health centres in New South Wales, Australia. Sex Health 2015. http://dx.doi.org/10.1071/SH15007. 10.1071/SH15007 26210444

[23] Graham S, Wand HC, Kaldor JM, O’Brien C, McCowen D, Soderlund C, et al. A sexual health intervention increases chlamydia and gonorrhoea testing in regional Aboriginal Community Controlled Health Services in New South Wales. Australiasian Sexual Health Conference, Darwin Australia, 2013. https://secure.ashm.org.au/ei/viewpdf.esp?id=119&file=c%3A%5CCertain%5CEventwin%5Cdocs%5Cpdf%5Cca13aAbstract00364%2Epdf. Accessed 20 April 2015.

[24] Graham S, Wand HC, Ward J, McCowen D, O’Brien C, Soderlund C, et al. Sustainability of a sexual health quality improvement program in regional Aboriginal Community Controlled Health Services in New South Wales. Australasian Sexual Health Conference. 2014. Sydney, Australia. https://secure.ashm.org.au/ei/viewpdf.esp?id=119&file=c%3A%5CCertain%5CEventwin%5Cdocs%5Cpdf%5Cca13aAbstract00364%2Epdf. Accessed 20 April 2015.

[25] Ward J, McGregor S, Guy RJ, Rumbold AR, Garton L, Silver BJ (2013). STI in remote communities: improved and enhanced primary health care (STRIVE) study protocol: a cluster randomised controlled trial comparing ‘usual practice’ STI care to enhanced care in remote primary health care services in Australia. BMC Infect Dis.

[26] National Health and Medical Research Council. Values and Ethics in Aboriginal and Torres Strait Islander Health Research. Guidelines for Ethical Conduct in Aboriginal and Torres Strait Islander Health Research. National Health and Medical Research Council. 2003. https://www.nhmrc.gov.au/_files_nhmrc/publications/attachments/e52.pdf. Accessed 20 April 2015.

[27] Eschiti V, Burhansstipanov L, Watanabe-Galloway S (1998). Culturally relevant ”navigator”patient support the native sisters. Cancer Pract.

[28] Burhansstipanov L, Wound DB, Capelouto N, Goldfarb F, Harjo L, Hatathlie L (1998). Culturally relevant “navigator” patient support. The native sisters. Cancer Pract.

[29] Boyle DIR, Kong F (2011). A systematic mechanism for the collection and interpetation of display format pathology test results from Australian primary care records. EJHI).

[30] Ward J, Bryant J, Worth H, Hull P, Solar S, Bailey S (2013). Use of health services for sexually transmitted and blood-borne viral infections by young Aboriginal people in New South Wales. Aust J Prim Health.

[31] Ward J, Goller J, Ali H, Bowring A, Couzos S, Saunders M (2014). Chlamydia among Australian Aboriginal and/or Torres Strait Islander people attending sexual health services, general practices and Aboriginal community controlled health services. BMC Health Serv Res.

[32] National Aboriginal Community Controlled Health Organisation and the Royal Australian College of General Practitioners: Sexual health and bloodborne viruses. National guide to a preventative health assessment for Aboriginal and Torres Strait Islander people, 2nd edition. 2012. In.: National Aboriginal Community Controlled Health Organisation and the Royal Australian College of General Practitioners. Accessed on 28 May 2015. http://www.naccho.org.au/download/aboriginal-health/1.National%20guide%20to%20a%20preventive%20health%20assessment%20for%20Aboriginal%20and%20Torres%20Strait%20Islander%20people%20(2).pdf.

[33] Commonwealth of Australia’s Department of Health and Ageing. Aboriginal and Torres Strait Islander health assessments (MBS item 715). Closing the Gap: Tackling Indigenous chronic disease. http://www9.health.gov.au/mbs/fullDisplay.cfm?type=note&q=A33&qt=noteID&criteria=715. Accessed on 21 April 2015.

[34] Silver BJ, Guy RJ, Wand H, Ward J, Rumbold AR, Fairley CK (2015). Incidence of curable sexually transmissible infections among adolescents and young adults in remote Australian Aboriginal communities: analysis of longitudinal clinical service data. Sex Transm Infect.

[35] Huang RL, Torzillo PJ, Hammond VA, Coulter ST, Kirby AC (2008). Epidemiology of sexually transmitted infections on the Anangu Pitjantjatjara Yankunytjatjara Lands: results of a comprehensive control program. Med J Aust.

[36] Hocking J, Low N, Guy R, Law M, Donovan B, Temple-Smith M, et al. on behalf of the ACCEPt Consortium. 2013. Protocol 12 PRT 09010: Australian Chlamydia Control Effectiveness Pilot (ACCEPt): a cluster randomised controlled trial of chlamydia testing in general practice. Lancet. 2013. http://www.thelancet.com/protocol-reviews/12PRT-9010.

